# Quantitative Ratings and Narrative Comments on Swiss Physician Rating Websites: Frequency Analysis

**DOI:** 10.2196/13816

**Published:** 2019-07-26

**Authors:** Stuart McLennan

**Affiliations:** 1 Institute of History and Ethics in Medicine Technical University of Munich Munich Germany; 2 Institute for Biomedical Ethics University of Basel Basel Switzerland

**Keywords:** physician rating websites, patient satisfaction, Switzerland

## Abstract

**Background:**

Physician rating websites (PRWs) have been developed as part of a wider move toward transparency around health care quality, and these allow patients to anonymously rate, comment, and discuss physicians’ quality on the Web. The first Swiss PRWs were established in 2008, at the same time as many international PRWs. However, there has been limited research conducted on PRWs in Switzerland to date. International research has indicated that a key shortcoming of PRWs is that they have an insufficient number of ratings.

**Objective:**

The aim of this study was to examine the frequency of quantitative ratings and narrative comments on the Swiss PRWs.

**Methods:**

In November 2017, a random stratified sample of 966 physicians was generated from the regions of Zürich and Geneva. Every selected physician was searched for on 4 rating websites (OkDoc, DocApp, Medicosearch, and Google) between November 2017 and July 2018. It was recorded whether the physician could be identified, what the physician’s quantitative rating was, and whether the physician had received narrative comments. In addition, Alexa Internet was used to examine the number of visitors to the PRWs, compared with other websites.

**Results:**

Overall, the portion of physicians able to be identified on the PRWs ranged from 42.4% (410/966) on OkDoc to 87.3% (843/966) on DocApp. Of the identifiable physicians, only a few of the selected physicians had been rated quantitatively (4.5% [38/843] on DocApp to 49.8% [273/548] on Google) or received narrative comments (4.5% [38/843] on DocApp to 31.2% [171/548] on Google) at least once. Rated physicians also had, on average, a low number of quantitative ratings (1.47 ratings on OkDoc to 3.74 rating on Google) and narrative comments (1.23 comment on OkDoc to 3.03 comments on Google). All 3 websites allowing ratings used the same rating scale (1-5 stars) and had a very positive average rating: DocApp (4.71), Medicosearch (4.69), and Google (4.41). There were significant differences among the PRWs (with the majority of ratings being posted on Google in past 2 years) and regions (with physicians in Zurich more likely to have been rated and have more ratings on average). Only Google (position 1) and Medicosearch (position 8358) are placed among the top 10,000 visited websites in Switzerland.

**Conclusions:**

It appears that this is the first time Google has been included in a study examining physician ratings internationally and it is noticeable how Google has had substantially more ratings than the 3 dedicated PRWs in Switzerland over the past 2 and a half years. Overall, this study indicates that Swiss PRWs are not yet a reliable source of unbiased information regarding patient experiences and satisfaction with Swiss physicians; many selected physicians were unable to be identified, only a few physicians had been rated, and the ratings posted were overwhelmingly positive.

## Introduction

Physician rating websites (PRWs) have been developed in many countries as part of a wider move toward transparency around health care quality, and these allow patients to anonymously rate, comment, and discuss physicians’ quality on the Web [1]. Switzerland has been no exception, with the first PRWs in Switzerland, OkDoc and Medicosearch, being established in 2008, at the same time as many international PRWs.

One of the key goals of PRWs is to improve patient welfare through influencing patient decision making by increasing the chance that patients will choose better quality physicians and benefit from this [2] and driving quality improvement by identifying aspects of care needing improvement, so that changes can be made in practice [2]. A related goal of PRWs is to improve patient health literacy to encourage and, therefore, respect patient autonomy [2]. Although recent research suggests that PRWs can influence patient decision making and have an impact on quality improvement [3,4], the ability of PRWs to achieve these goals is likely limited without sufficient number of ratings, as without enough ratings the resulting information is unlikely to be fair for the rated physicians or useful to users of PRWs [5-7].

A recent systematic search of PRWs internationally found that the majority of PRWs were registered in the United States and Germany [8], and the majority of the previous research on PRWs came from these 2 countries. This research has highlighted some key shortcomings of PRWs, including incomplete lists of physicians, low number of physicians rated, and low number of ratings per physician that are overwhelmingly positive. In the United States, Black et al reported in 2009 that their analysis of 6703 ratings of 6101 providers on the PRW RateMDs found that the average number of ratings per physician was 2.7, and their analyses of narrative comments found more positive than negative terms [9]. In 2010, Lagu et al reported that the portion of physicians from a sample of 300 Boston-based physicians that could be identified on 33 websites ranged from 0% to 90.7%; 27% of the sample had been rated once, the average number of ratings per physician was 1.4, and in total there were 190 reviews—170 reviews included quantitative ratings (88% positive), and 66 reviews included narrative comments (89% positive) [5]. In 2011, Kadry et al reported that their analysis of 4999 ratings on PRWs found that physician ratings were mostly positive on all the different rating scales used by PRWs (average of 77 on a 100-point scale, 3.84 on a 5-point scale, and 3.1 on a 4-point scale) [10]. In 2012, Gao et al reported that 16% of the physicians were rated on the PRW RateMDs between 2005 and 2010 [11], the average number of ratings per physician was 3.2, and the ratings were generally positive (mean 3.93 on a 5-point scale). In 2013, Ellimoottil et al reported that in a random sample of 500 urologists [12], 79.6% the of physicians were rated at least once on 10 websites, the average number of reviews per physician was 2.4, 86% of the physicians had a positive rating, and 45% of the physicians had a narrative comment (75% of which were very positive, positive, or neutral). In 2014, Sobin and Goyal reported that in their sample of 281 otolaryngologists, 94.7% could be identified on Healthgrades and 87.9% could be identified on Vitals; of those who were identifiable, 69.9% had been rated at least once on Healthgrades and 81.8% on Vitals, and the average rating was 4.4 on Healthgrades (5-point scale) and 3.4 on Vitals (4-point scale) [13]. In 2017, Murphy et al reported that their analysis of the impact of physician probation on ratings found that average number of ratings per physician was 5.2 for physicians on probation and 4 for controls on Vitals, Healthgrades, and RateMDs, and the average rating on a 5-point scale for physicians on probation was 3.7 compared with 4.0 for controls [14].

In Germany, Strech and Reimann reported in 2012 that from a sample of 298 physicians from Hamburg and Thuringia, 75% to 98% of the physicians could be identified on 6 PRWs, 3% to 26% of the physicians had been rated at least once, the average number of ratings per physician ranged from 1.1 to 3.1, and the average converted standardized rating (1=positive, 2=neutral, and 3=negative) ranged from 1.1 to 1.5 [15]. In 2013, Emmert et al reported that their analysis of 127,192 ratings from 2012 on the German PRW jameda found that 37% of the physicians had been rated, rated physicians had an average of 2.37 ratings, and almost 80% of all ratings were from the 2 best rating categories [16]. In 2014, Emmert et al reported that in their sample of 106 physicians, 96% could be identified on 5 PRWs, 50% of the physicians had been rated at least once, there was an average of 3.08 ratings per physician, and 86% of the ratings were positive (with 75% assigned to the best rating category and only 5% to the worst category) [17]. In 2017, McLennan et al reported that their update study using a sample of 298 physicians from Hamburg and Thuringia found that 65.1% to 94.6% of the physicians could be identified on 6 PRWs, 16% to 83% of the sample had been rated at least once, the average number of ratings per physician ranged from 1.2 to 7.5, and the average converted standardized rating (1=positive, 2=neutral, and 3=negative) ranged from 1.0 to 1.2 [18].

In recent years, there has also been an increasing number of studies published regarding PRWs in China [19-24]. Regarding the frequency of ratings, Hao reported in 2015 that an analysis of the PRW Good Doctor found that 112,873 physicians had received 731,543 quantitative and 772,979 qualitative reviews, on average 37% of the physicians had been reviewed, and the majority of the quantitative reviews were positive (88% positive for treatment effect and 91% positive for bedside manner) [23]. There have also been studies examining the frequency of ratings in other countries. For instance, Liu et al reported in 2018 that their analysis of 640,603 ratings for 57,412 Canadian physicians found that the average number of ratings per physician was 11.2, and the ratings were generally positive with an average of 3.9 (5-point scale) [25]. In 2012, Greaves et al also reported that their analysis of ratings of family practices posted on National Health Service Choices website in the United Kingdom found that 61% of the practices had been rated, and the average number of ratings per practice was 2.1 [26].

Switzerland is a Central European country with a population of about 8.4 million people and 4 official languages (German, French, Italian, and Romansh). The Swiss health care system is highly complex and decentralized, organized around 3 levels of Swiss government (the federal, the cantonal, and the municipalities) [27,28]. All Swiss residents are required to purchase basic mandatory health insurance that is offered by competing nonprofit insurers. Mandatory health insurance covers most general practitioner (GP) and specialist services (among other things), and people not enrolled in managed care plans generally have free choice of professionals. In addition, for-profit insurers offer private complementary insurance for services not covered by mandatory health insurance. Ambulatory physicians (including GPs and specialists) are typically reimbursed in accordance with a standardized fee schedule known as TARMED [27,28].

Although the first PRWs in Switzerland were launched in 2008, there has been limited research conducted on PRWs in Switzerland to date [8,29,30]. Swiss PRWs, however, operate in a rather unique regulatory environment. Owing to Switzerland’s restrictive legal framework for data protection, a federal data commissioner decided that negative comments had to be removed from OkDoc, which now only acts as a recommendation portal and explicitly states that any negative comment will be deleted (“Only positive comments recommending your doctor will be accepted. Any negative post will be deleted. Thank you for respecting okdoc’s principles!” [author translation]). Although Medicosearch allows negative comments, it informs the concerned physician before publishing it on the Web, so that the physician can decide if the negative feedback is activated. However, if the physician refuses, the feedback function is deactivated, removing also the positive comments [31]. This situation is in stark contrast to more liberal systems (eg, the Federal Court of Justice of Germany confirmed in 2014 the permissibility of ratings on the basis of the right to freedom of expression [32-34]) and likely has important implications in relation to the frequency of ratings and how negative comments are handled on Swiss PRWs.

This study, therefore, examined the frequency of quantitative ratings and narrative comments on Swiss PRWs. In particular, it aimed to explore (1) the number of identifiable physicians on Swiss PRWs, (2) the proportion of physicians with ratings or comments on Swiss PRWs, (3) the average and the maximum number of ratings or comments per physician on Swiss PRWs, (4) the average rating on Swiss PRWs, (5) the website visitor ranking positions of Swiss PRWs, and (6) provide baseline results for future research to assess the development of Swiss PRWs. It is important to examine these issues to help inform future research and health policy in Switzerland in relation to PRWs.

## Methods

### Sample

A random stratified sample of 966 physicians was generated from the regions of Zürich and Geneva. Zürich is the largest city in Switzerland with a total population of 402,762 (December 2016) [35] and is located in north-central Switzerland. Geneva is the second largest city in Switzerland with a total population of 198,979 (December 2016) [35] and is located in south-western Switzerland. The regions of Zürich and Geneva were chosen because of language (German vs French) and comparable number of total physicians (Zürich 3254 physicians and Geneva 2780 physicians) considerations.

In November 2017, all physicians in these regions, working in general practice, obstetrics and gynecology, pediatrics, and dermatology and venereology, were searched for on the Swiss Medical Association's (FMH) medical registry (Ärzteverzeichnis). Specialties were primarily selected based on previous research [15,18]. From each region, a random sample was generated for each specialty based on a 95% confidence level and 5% confidence interval. From Zürich, the random sample comprised 254 of 747 general practice physicians, 85 of 109 obstetrics and gynecology physicians, 74 of 92 pediatrics physicians, and 53 of 61 dermatology and venereology physicians. Therefore, the Zürich sample of 466 physicians represents 46.2% of a total of 1009 physicians. From Geneva, the random sample comprised 272 of 930 general practice physicians, 86 of 111 obstetrics and gynecology physicians, 96 of 128 pediatrics physicians, and 46 of 52 dermatology and venereology physicians. Therefore, the Geneva sample of 500 physicians represents 40.9% of a total of 1221 physicians (see Table 1).

**Table 1:** Physician samples per region.

Specialty	Zurich		Geneva		Total	
	Total physicians found, N	Physicians selected for sample, n (%)	Total physicians found, N	Physicians selected for sample, n (%)	Total physicians found, N	Physicians selected for sample, n (%)
General practitioners	747	254 (34.0)	930	272 (29.2)	1677	526 (31.36)
Obstetrics and gynecology	109	85 (77.9)	111	86 (77.5)	220	171 (77.7)
Pediatrics	92	74 (80.4)	128	96 (75.0)	220	170 (77.3)
Dermatology and venereology	61	53 (86.8)	52	46 (88.5)	113	99 (87.6)
Total	1009	466 (46.18)	1221	500 (40.95)	2230	966 (43.32)

### Data Collection

To identify PRWs on which patients can rate and review physicians in Switzerland, a systematic Web-based search was conducted in June 2016 from a patient´s perspective. A total of 10 key search words (see Table 2) in the German language were identified from previously published studies on PRWs conducted in Germany [15,36,37]. As most internet users use a search engine to find health information [36], the systematic search was conducted on Google, which is the most visited search engine in Switzerland, with a market share reported to be 93.5% (Alexa data valid of May 24, 2016). Each search term was searched for and the first 50 hits (5 pages) were examined. As 70% of users only look at the first 2 result pages or less [36], this approach reflects the search behavior of most users. A total of 500 hits were, therefore, examined. A website was included if it allowed users to view quantitative ratings and/or narrative comments about Swiss physicians in a structured manner without having to open an account or log onto the website. Websites that were not dedicated to Swiss physicians were excluded. A total of 3 PRWs were included: OkDoc (found by 8/10 of the search terms), DocApp (found by 4/10 of the search terms), and Medicosearch (found by 2/10 of the search terms). In addition, Google itself allows users to rate and comment on physicians via Google reviews. Furthermore, although the health care information portal doktor does not provide the option for ratings, it links to Google reviews. Google was, therefore, also included in the study, and as far as the author is aware, this is the first time Google has been included in a study examining physician ratings internationally.

Selected physicians were, therefore, searched for on a total of 4 websites: OkDoc, DocApp, Medicosearch, and Google. On each website, every selected physician was searched for between November 2017 and July 2018, and it was recorded in a SPSS file whether the physician could be found, the physician’s rating, the number of ratings and narrative comments, and the text of narrative comments. As OkDoc now only allows recommendations, the number of these recommendations were assigned to the *number of ratings*. All websites allowing ratings (DocApp, Medicosearch, and Google) used the same rating scale (1-5 stars); a rating of 4 to 5 stars was considered a positive rating, 3 stars a neutral rating, and 1 to 2 stars a negative rating.

Alexa Internet was used to examine the number of visitors to PRWs, compared with other websites. Founded in 1996, Alexa provides commercial Web traffic data and analytics. Traffic estimates are based on data from a global traffic panel and from websites that have chosen to install the Alexa script on their site and certify their metrics. The Alexa global traffic ranking is based on the estimated average of daily unique visitors and its estimated number of page views over the past 3 months relative to all other websites. In addition, Alexa provides a similar country-specific ranking based on how a website ranks relative to other websites in a particular country over the past month. The PRWs were searched for on Alexa in November 2017 and their Switzerland specific ranking was recorded.

**Table 2:** Systematic search of the Swiss physician rating websites on Google.

Number	Search terms in German	English translation	Physician rating website found
1	Arztsuche	Physician search	None
2	Arzt finden	Find a physician	OkDoc, DocApp
3	Arzt bewerten	Rate my physician	OkDoc, Medicosearch, DocApp
4	Arztbewertung	Physician rating	OkDoc, DocApp
5	Arzt empfehlen	Recommend a physician	OkDoc
6	Arztempfehlung	Physician recommendation	None
7	Ärzte Beurteilungen‎	Physician reviews	OkDoc
8	Online Arztbewertung	Online physician rating	OkDoc, DocApp, Medicosearch
9	Arztbewertungsportal	Physician rating website	OkDoc
10	Guter Arzt	Good physician	OkDoc

### Data Analysis

Descriptive statistics included means and standard deviations for continuous variables and percentages for categorical variables. To analyze whether differences exist within an individual PRW as well as across PRWs between the 2 regions (Zürich and Geneva) and between GPs and specialists (obstetrics and gynecology, pediatrics, and dermatology and venereology), chi-squared tests were used for categorical data and *t* tests for continuously distributed data. To analyze differences across the PRWs, a *sum score* was created; for example, in relation to how many physicians were identified on PRWs, a score ranging from 0 (not identified on any PRW) to 4 (identified on all PRWs) was created and, subsequently, it was analyzed whether the mean of this score was different between the 2 groups being examined. All analyses were performed with a significance level alpha set to .05 and 2-tailed tests, using Statistical Package for the Social Sciences (SPSS version 24 for Windows, IBM Corporation).

## Results

The full results regarding the quantitative ratings and narrative comments are presented in Tables 3-5. See Multimedia Appendix 1 for the full results of comparisons between the 2 regions and Multimedia Appendix 2 for the results of comparisons between GPs and specialists.

**Table 3:** Quantitative rating.

Region, website		Physicians, n (%)		Ratings per physician		Rating, mean (SD)
		Identifiable	Rated^a^	Mean (SD)	Maximum	
**Zurich (N=466)**						
	OkDoc	225 (48.3)	35 (15.5)	1.26 (0.6)	4	—^b^
	DocApp	406 (87.1)	37 (9.1)	2.38 (5.2)	32	4.70 (0.7)
	Medicosearch	356 (76.4)	74 (20.7)	2.78 (5.3)	32	4.68 (0.8)
	Google	268 (57.5)	150 (55.9)	4.56 (5.9)	56	4.38 (0.9)
**Geneva (N=500)**						
	OkDoc	185 (37.0)	41 (22.1)	1.66 (1.1)	6	—
	DocApp	437 (87.4)	1 (0.2)	1	1	5
	Medicosearch	331 (66.2)	22 (6.6)	1.23 (0.5)	3	4.73 (0.8)
	Google	280 (56.0)	123 (43.9)	2.67 (2.2)	13	4.45 (0.9)
**Overall (N=966)**						
	OkDoc	410 (42.4)	76 (18.5)	1.47 (0.9)	6	—
	DocApp	843 (87.3)	38 (4.5)	2.34 (5.1)	32	4.71 (0.7)
	Medicosearch	687 (71.1)	96 (13.9)	2.42 (4.7)	32	4.69 (0.8)
	Google	548 (56.7)	273 (49.8)	3.74 (4.7)	56	4.41 (0.9)

^a^Each n value is a sample from the identifiable physician population value.

^b^Data not applicable.

**Table 4:** Narrative comments.

Region, website		Physicians, n (%)		Comments per physician	
		Identifiable	With comments	Mean (SD)	Maximum
**Zurich (N=466)**					
	OkDoc	225 (48.3)	18 (8.0)	1.11 (0.3)	2
	DocApp	406 (87.1)	37 (9.1)	2.38 (5.2)	32
	Medicosearch	356 (76.4)	74 (20.7)	2.78 (5.3)	32
	Google	268 (57.5)	104 (38.8)	3.7 (5.5)	49
**Geneva (N=500)**					
	OkDoc	185 (37.0)	13 (7.0)	1.38 (0.7)	3
	DocApp	437 (87.4)	1 (0.2)	1	1
	Medicosearch	331 (66.2)	22 (6.6)	1.27 (0.6)	3
	Google	280 (56.0)	67 (23.9)	2.0 (2.1)	12
**Overall (N=966)**					
	OkDoc	410 (42.4)	31 (7.5)	1.23 (0.5)	3
	DocApp	843 (87.3)	38 (4.5)	2.34 (5.1)	32
	Medicosearch	687 (71.1)	96 (13.9)	2.44 (4.7)	32
	Google	548 (56.7)	171 (31.2)	3.04 (4.6)	49

**Table 5:** Distribution of narrative comments.

Region, website		Total, N	Distribution of comments, n (%)									
			2018 (half year)	2017	2016	2015	2014	2013	2012	2011	2010	2009	2008
**Zurich**													
	OkDoc	20	0	0	1 (5)	0	0	0	0	2 (10)	2 (10)	3 (15)	12 (60)
	DocApp	56	3 (5)	22 (39)	24 (43)	7 (13)	0	0	0	0	0	0	0
	Medicosearch	206	6 (2.9)	57 (27.6)	59 (28.6)	6 (2.9)	8 (3.8)	11 (5.3)	16 (7.8)	18 (8.7)	12 (4.9)	12 (5.8)	1 (0.5)
	Google	386	160 (41.4)	187 (48.4)	29 (7.5)	5 (1.2)	1 (0.2)	3 (0.7)	0	0	1 (0.2)	0	0
	Total	668	169 (25.2)	266 (39.8)	113 (16.9)	18 (2.6)	9 (1.3)	14 (2.0)	16 (2.3)	20 (2.9)	15 (2.2)	15 (2.2)	13 (1.9)
**Geneva**													
	OkDoc	18	0	0	0	0	0	0	0	1 (6)	0	3 (17)	14 (78)
	DocApp	1	0	1 (100)	0	0	0	0	0	0	0	0	0
	Medicosearch	28	0	0	2 (7)	0	2 (7)	2 (7)	1 (4)	5 (18)	7 (25)	9 (32)	0
	Google	134	75 (55.9)	39 (29.1)	12 (8.9)	2 (1.5)	4 (2.9)	2 (1.5)	0	0	0	0	0
	Total	181	75 (41.4)	40 (22.0)	14 (7.7)	2 (1.1)	6 (3.3)	4 (2.2)	1 (0.5)	6 (3.3)	7 (3.8)	12 (6.6)	14 (7.7)
**Overall**													
	OkDoc	38	0	0	1 (3)	0	0	0	0	3 (8)	2 (5)	6 (16)	26 (68)
	DocApp	57	3 (5)	23 (40)	24 (42)	7 (12)	0	0	0	0	0	0	0
	Medicosearch	234	6 (2.5)	57 (24.3)	61 (26)	6 (2.5)	10 (4.2)	13 (5.5)	17 (7.2)	23 (9.8)	19 (8.1)	21 (8.9)	1 (0.4)
	Google	520	235 (45.1)	226 (43.4)	41 (7.8)	7 (1.3)	5 (0.1)	5 (0.1)	0	0	1 (0.1)	0	0
	Total	849	244 (28.7)	306 (36)	127 (14.9)	20 (2.3)	15 (1.7)	18 (2.1)	17 (2.0)	26 (3.0)	22 (2.5)	27 (3.1)	27 (3.1)

### Quantitative Ratings

#### Identifiable Physicians

Overall, the portion of physicians from the random sample that were able to be identified on the selected PRWs ranged from 42.4% (410/966) on OkDoc to 87.3% (843/966) on DocApp. Physicians were identified significantly more in Zurich on OkDoc (X^2^_1_=12.6; *P*<.001) and Medicosearch (X^2^_1_=12.2, *P*<.001). Across all PRWs, there was also a significant difference between Zurich (mean 2.7; SD 1.2) and Geneva (mean 2.5; SD 1.2) (*t*_964_=2.9; *P*=.004). GPs were identified significantly more than specialists on Medicosearch (X^2^_1_=7.3; *P*=.007).

#### Rated Physicians

Overall, of the physicians identified, the portion that had been rated at least once ranged from 4.5% (38/843) on DocApp to 49.8% (273/548) on Google. Physicians from Zurich were rated significantly more on DocApp (X^2^_1_=38.6; *P*<.001), Medicosearch (X^2^_1_=28.5; *P*<.001), and Google (X^2^_1_=7.9; *P*=.005). Across all PRWs, there was also a significant difference between Zurich (mean 1.0; SD 0.9) and Geneva (mean 0.8; SD 0.8) (*t*_274_=2.0; *P*=.046). GPs were rated significantly on Google (X^2^_1_=19.1; *P*<.001). Across all PRWs, there was also a significant difference between GPs (mean 0.9; SD 0.8) and specialists (mean 1.1; SD 0.9) (*t*_274_=−2.2; *P*=.03).

#### Average and Maximum Number of Ratings

Overall, the average number of ratings per physician ranged from 1.47 (SD 0.9) on OkDoc to 3.74 (SD 4.7) on Google. The maximum number of ratings per physician ranged from 6 on OkDoc to 56 on Google. Whereas the physicians in Geneva (mean 1.6; SD 1.1) had significantly more ratings on average than the physicians in Zurich (mean 1.3; SD 0.6) on OkDoc (*t*_65_=2.1; *P*=.04), the physicians in Zurich had significantly more ratings on average than the physicians in Geneva on Medicosearch (mean 2.8, SD 5.3 vs mean 1.2, SD 0.5; *t*_77_=2.5; *P*=.02) and Google (mean 4.6, SD 5.9 vs mean 2.7, SD 2.2; *t*_198_=3.7; *P*<.001). Similarly, whereas GPs (mean 1.7; SD 1.1) had significantly more ratings on average than specialists (mean 1.3; SD 0.6) on OkDoc (*t*_57_=2.1; *P*=.04), specialists (mean 4.5; SD 5.5) had significantly more ratings on average than GPs (mean 2.8; SD 3.2) on Google (*t*_249_=−3.2; *P*=.001).

#### Average Rating

Overall, the 3 websites allowing ratings all used the same rating scale (1-5 stars) and had a very positive average rating: DocApp, 4.71; Medicosearch, 4.69; and Google, 4.41. There were no significant differences between the regions or between GPs and specialists.

### Narrative Comments

#### Physicians With Comments

Overall, of the physicians identified, the portion that had received at least 1 comment ranged from 4.5% (38/843) on DocApp to 31.2% (171/548) on Google. Physicians from Zurich had received a comment significantly more often than Geneva physicians on DocApp (X^2^_1_=38.2; *P*<.001) and Google (X^2^_1_=14.8; *P*<.001). GPs also had received a comment significantly more often than specialists on Google (X^2^_1_=23.1; *P*<.001).

#### Average and Maximum Number of Comments

Overall, the average number of comments per physician ranged from 1.23 (SD 0.5) on OkDoc to 3.04 (SD 4.6) on Google. The maximum number of comments per physician ranged from 3 on OkDoc to 49 on Google. Physicians from Zurich had significantly more comments on average than physicians in Geneva on Medicosearch (mean 2.8, SD 5.3 vs mean 1.3, SD 0.6; *t*_77_=2.4; *P*=.02) and Google (mean 3.7, SD 5.6 vs mean 2.0, SD 2.1; *t*_142_=2.9; *P*=.005). There were no significant differences between GPs and specialists.

#### Distribution of Comments

**Figure 1:**
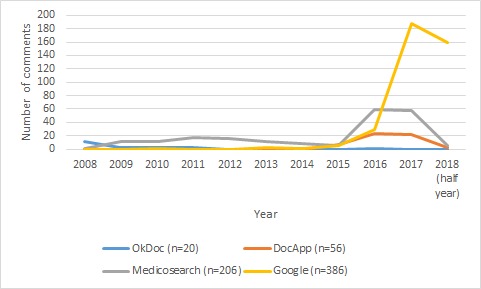
Distribution of comments in Zurich.

**Figure 2:**
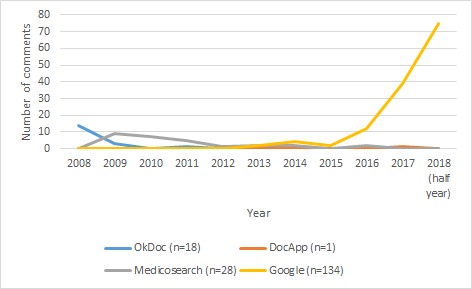
Distribution of comments in Geneva.

**Figure 3:**
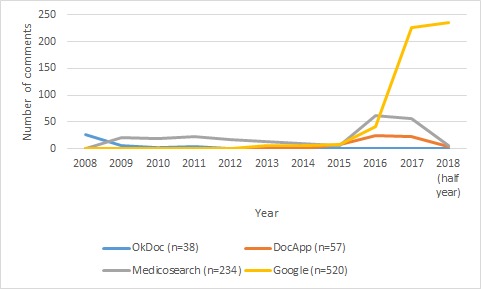
Distribution of comments in overall.

Overall, the selected physicians in the sample had a total number of 849 comments from 2008 to 2018 (half year), with 80% of comments (677/849) having been posted during the last 2 and a half years (2016 to 2018). The majority of comments in Zurich (386/668, 57.7%) and Geneva (134/181, 74.0%) were made on Google. OkDoc only had 1 comment posted for all 966 physicians in the sample during the last 5 and a half years (2012-2018). Physicians in the Zurich sample also had substantially more comments (668 comments) compared with physicians in the Geneva sample (181 comments), with 78.7% (668/849) of total comments coming from physicians in Zurich (see Figures 1-3).

### Website Visitor Ranking Positions

Whereas Google was in position 1 on Alexa for the most visited websites in Switzerland, Alexa indicated that the use of the dedicated PRWs was not common, with only Medicosearch (position 8358) placed among the top 10,000 visited websites in Switzerland. DocApp was ranked 19,858, whereas there were insufficient data for OkDoc. In comparison, the hotel rating site TripAdvisor ranked 154. Rankings are as of November 10, 2017.

## Discussion

### Principal Findings

This is the first study to examine the frequency of ratings on Swiss PRWs and it has resulted in a number of key findings: (1) many of the selected physicians could not be identified on Swiss PRWs, (2) very few of the selected physicians had been rated quantitatively or qualitatively and those who had been rated had on average a low number of ratings, (3) there were significant differences among the PRWs, with Google having substantially more ratings in the past 2 and a half years than the 3 dedicated PRWs, (4) there were also significant differences between regions, with physicians in Zurich more likely to have been rated and have more ratings on average, and (5) all 3 websites allowing ratings had a very positive average rating overall.

#### Identifiable Physicians

Incomplete lists of physicians have been identified as a weakness of many PRWs internationally [5,6], and it appears that the users of PRWs in Switzerland are also not able to find many physicians on Swiss PRWs, with the overall portion of selected physicians that was able to be identified ranging from 42.4% on OkDoc to 87.3% on DocApp. Although the result for OkDoc may not be overly surprising, as it appears to be rather inactive following the decision of a federal data commissioner that negative comments have to be removed [31], only 56.7% of the physicians could be identified on Google. This range is lower compared with the findings of studies on PRWs in other countries. For instance, a 2010 study in Germany found that the portion of physicians that could be identified on German PRWs ranged from 76% to 98% [15], whereas a follow-up study in 2014 found a range of 65% to 95% [18]. The current lack of comprehensiveness of Swiss PRWs could inhibit their usefulness, and it will be important to consider how more complete lists of physicians can be provided.

#### Number of Ratings

It is widely considered that a key factor in PRWs being successful in their goals of influencing patients’ decision making and driving quality improvement is having sufficient number of ratings [5,6]. However, low number of ratings has been identified as a key shortcoming of PRWs in many countries, which has called into question their representativeness, validity, and usefulness [5,6]. This study also indicates that insufficient rating can also be an issue for Swiss PRWs, with only a few of the identifiable physicians having been rated quantitatively (4.5% on DocApp to 49.8% on Google) or qualitatively (4.5% on DocApp to 31.2% on Google) at least once. Rated physicians also had on average a low number of quantitative ratings (1.47 ratings on OkDoc to 3.74 rating on Google) and narrative comments (1.23 comment on OkDoc to 3.03 comments on Google). Although the results of this study were lower than those found in a recent study in Germany, which found that 16% to 83% of the sample had been rated at least once and these physicians had an average number of ratings between 1.2 and 7.5 [18], they were very similar to the results of the previous studies in Germany [15-17] and the United States [5,9,11,12]. However, it should be noted that many of these studies’ reported figures were, unlike this study, portions of the total sample rather than a portion of the identifiable physicians and were, therefore, slightly higher than reported.

There is, however, currently limited research internationally examining the reasons why patients do not rate their physicians on PRWs. The use of PRWs first requires the public to be aware of them [38]. Recent studies in the United States and Germany suggest that a lack of awareness is no longer a key barrier to PRW usage in these countries [38-41], although a recent study in the England found public awareness of PRWs to still be very low [42]. However, despite the fact that awareness of PRWs is an important factor, it should be noted that although the studies conducted in the United States and Germany found high awareness of the PRWs, the level of PRW usage was still found to be comparable with previous studies [3,43], suggesting that even if awareness of PRWs increases, there are other factors behind the low level of physician ratings. A recent qualitative study in Germany aiming to examine these other factors identified 2 key overarching groups of factors—first, factors concerning the physician-patient relationship and second, factors regarding the technical aspects of PRWs [44]. Although a qualitative study in 2016 with participants residing in the German-speaking part of Switzerland also highlighted the need to improve the design of PRWs, the study involved German PRWs rather than Swiss PRWs [29]. Further research is, therefore, needed in Switzerland regarding public awareness of PRWs and factors influencing patients’ decision to rate or not rate physicians.

There were significant differences among the PRWs in relation to the frequency of ratings. Although OkDoc was the first Swiss PRWs, launched in 2008, it was evident how inactive the website had become since the federal data commissioner decided that negative comments had to be removed. Although the portion of the rated physicians on OkDoc is still higher than DocApp and Medicosearch, the distribution of comments indicates that this is because of ratings posted in 2008. Indeed, OkDoc only had 1 comment posted for all 966 physicians in the sample during the last 5 and a half years (2012-2018). This situation is possibly exacerbated by Okdoc being the only Swiss PRW not to offer an English version of the website, given the high number of foreign residents in Switzerland [45].

In contrast, it is noticeable how Google has had substantially more quantitative ratings and narrative comments than the 3 dedicated PRWs in the past 2 and a half years, and how it has been able to establish itself as the most used website in Switzerland for physician ratings. It remains to be seen whether the other dedicated PRWs will be able to increase their number of physician ratings in the future or whether Google will continue to dominate the market. Future updates will be helpful to assess how this develops. In the meantime, given the current large differences among the PRWs in terms of how many physicians can be identified and the number physician ratings, it would be advisable for the users of PRWs to utilize a number of PRWs when searching for a new physician.

There were also significant differences between the 2 regions (Zurich and Geneva) in relation to the frequency of ratings, with physicians from Zurich having been rated at least once more often and having on average more ratings. It is, however, unclear what the reason is behind these differences between Zurich and Geneva. These differences may simply reflect differences in the networks of the PRWs or may be a result of more cultural factors. Previous research in Switzerland has indicated that the German-speaking Swiss tend to be more critical toward their physicians and less-dependent on them, compared with the French- and the Italian-speaking Swiss [46,47]. However, further research is needed to examine what is causing these differences in the use of Swiss PRWs.

#### Average Rating

Although there have been concerns from the medical profession that PRWs would be primarily used for *doctor-bashing* [48,49], these fears have proved to be unfounded with the previous international research finding ratings on PRWs to be on average very positive [5,10-12,16-18]. This study has found a similar situation in Switzerland; the 3 PRWs that allow ratings all used the same rating scale (1-5 stars) and had a very positive average rating: DocApp, 4.71; Medicosearch, 4.69; and Google, 4.41. Such overwhelmingly positive ratings also raise concerns about the representativeness, validity, and usefulness of information on PRWs [5,6].

In Switzerland, it appears that the restrictive legal framework regarding data protection may be having a huge impact on the types of ratings that are on Swiss PRWs. As a key goal of PRWs is to promote transparency, this is concerning and suggests that Swiss PRWs are not a reliable source of unbiased information regarding patient experiences and satisfaction with Swiss physicians. Addressing the potential harms to physicians without limiting the potential health literacy benefits for patients is challenging; however, as Strech has noted: “In many countries the medical profession enjoys privileges such as strong advocacy groups and special social facilities. Thus, the denial of transparency on patient experiences and satisfaction (with physician performance) requires a strong rationale” [2]. Further consideration is needed to determine whether the current lack of transparency on Swiss PRWs is justified or whether changes are required.

### Limitations

This study has a number of limitations that should be taken into account when interpreting the results. First, although a systematic Web-based search of Swiss PRWs was conducted, there might be other types of websites that allow Swiss physicians to be rated, which were not included in this study. This is a fast-moving area and it does appear that there are some websites that have started allowing ratings or making ratings publicly available after this project commenced (eg, deindoktor and doctena), which should be added to any future studies examining PRWs in Switzerland. Second, only German search terms were used for the systematic Web-based search of Swiss PRWs. Although the author is confident that no important Swiss PRWs were missed at the time of developing and conducting the project, it would be preferable if French and Italian search terms are also included in future research in Switzerland to ensure that no PRWs are being missed. Third, the sample was only taken from 2 regions in Switzerland, which might have limited the generalizability of the results. Although the study used a representative random sample from a German- and a French-speaking region of Switzerland with comparable number of physicians, given the significant differences found between the 2 regions, it would be helpful for future research to include other regions to examine whether these differences can be found between other German- and French-speaking regions and in the Italian-speaking region of Ticino. Finally, because of practical considerations of searching for 966 physicians on 4 different websites, data were collected over a 9-month period. This might have had led to differences among the PRWs that were examined at the beginning of the data collection compared with those PRWs examined at the end of data collection.

### Conclusions

With a growing number of patients utilizing the internet in relation to their health care [50], it is expected that PRWs will play an increasingly important role in selecting a new physician. However, for PRWs to be helpful for the users, and fair for the rated physicians, it is important that PRWs have a sufficient number of ratings. This study indicates that Swiss PRWs are currently not an effective mechanism of collecting patient experiences as a source of information for others; many physicians could not be identified, of the physicians identified, most had not been rated, and those that had been rated had on average only a few ratings. However, there were significant differences among the PRWs. As far as the author is aware, this is the first time Google has been included in a study examining physician ratings internationally and it is noticeable how Google has had substantially more ratings than the 3 dedicated Swiss PRWs in the past 2 and a half years. This is an important development not previously reported in the context of public reporting activities. Given Google’s general market dominance globally, Google might become the primary website for physician rating and it will be important for health systems to reflect on the implications of this. However, in the meantime, given the current large differences among the PRWs, it would be advisable for the users of PRWs to utilize a number of PRWs when searching for a new physician [18]. However, in addition to the low number of ratings, the ratings that are on Swiss PRWs are overwhelmingly positive, which suggests that Swiss PRWs are also not a reliable source of unbiased information regarding patient experiences and satisfaction with Swiss physicians. Although more research is needed to examine the factors influencing the low number of ratings and the lack of negative ratings in Switzerland, it appears that Switzerland’s restrictive legal framework regarding data protection may be a key factor. There is a need for more consideration to be given to the correct equilibrium between protecting physicians from harm and promoting patients’ autonomy and health literacy. However, as long as the current restrictive legal framework remains, the utility of Swiss PRWs is likely to be weakened from the patient’s point of view. Swiss PRWs should seek to enrich the utility of their websites with additional features, such as the possibility to book an appointment with a physician through the PRW (as already offered by Medicosearch and other websites, such as deindoktor and doktena) and providing information about the language spoken by the specific physician (as already offered by DocApp and Medicosearch). Such features may increase the utility of the PRWs and perhaps also help increase the number of physician ratings in the long term (eg, by sending invites to patients after the appointments they have booked on the Web).

## Appendix

Multimedia Appendix 1 Results of comparisons between regions.

Multimedia Appendix 2 Results of comparisons between specialities.

## Abbreviations

GP: general practitioner
PRW: physician rating website
