# Supplementary material for: Quantitative Ratings and Narrative Comments on Swiss Physician Rating Websites: Frequency Analysis
Source: J Med Internet Res. 2019 Jul 26;21(7):e13816. doi: 10.2196/13816 (PMC6688440; doi:10.2196/13816)
Supplement: Multimedia Appendix 1 [file jmir_v21i7e13816_app1.pdf]

Multimedia Appendix 1. Results of comparisons between regions

Comparison	okdoc	docapp	medicosearch	google.ch	Overall
Quantitative ratings					
Identifiable physicians					
1. Zurich (%)	**1.225/466 (48.3)**	1. 406/466 (87.1)	**1.356/466 (76.4)**	1.268/466 (57.5)	**1.M=2.7,SD=1.2**
2. Geneva (%)	**2.185/500 (37.0)**	2. 437/500 (87.4)	**2.331/500 (66.2)**	2. 280/500 (56)	**2.M=2.5,SD=1.2**
Chi-squared-test / T-test	**$\chi^2_{(1)}$=12.6, *P*<.001**	$\chi^2_{(1)}$=.02, *P*=.90	**$\chi^2_{(1)}$=12.2, *P*<.001**	$\chi^2_{(1)}$=.2, *P*=.64	**t(964)=2.9, *P*=.004. 95% CI: .073,.381**
Rated physicians					
1. Zurich	1. 35/225 (15.5)	**1. 37/406 (9.1)**	**1. 74/356 (20.7)**	**1.150/268 (55.9)**	**1. M=1.0, SD=.9**
2. Geneva	2. 41/185 (22.1)	**2. 1/437 (0.2)**	**2. 22/331 (6.6)**	**2.123/280 (43.9)**	**2. M=.8, SD=.8**
Chi-squared-test / T-test	$\chi^2_{(1)}$=2.9, *P*=.09	**$\chi^2_{(1)}$=38.6, *P*<.001**	**$\chi^2_{(1)}$=28.5, *P*<.001**	**$\chi^2_{(1)}$=7.9, *P*=.005**	**t(274)=2.0, *P*=.046. 95% CI: .004,.401**
Average number of ratings					
1. Zurich	**1.M=1.3,SD=.6**	1.M=2.2,SD=5.2	**1.M=2.8,SD=5.3**	**1.M=4.6,SD=5.9**	[1]
2. Geneva	**2.M=1.7,SD=1.1**	2.M=1.0,SD=NA	**2.M=1.2,SD=.5**	**2.M=2.7,SD=2.2**	
T-test	**t(65)=-2.1, *P*=.04. 95% CI: -.792,.011**	t(36)=.3, *P*=.795. 95% CI: -9.327,12.084	**t(77)=2.5, *P*=.02. 95% CI: .317,2.796**	**t(198)=3.7, *P*<.001. 95% CI: .869,2.907**	
Average rating					
1. Zurich	NA	1.M=4.7, SD=.7	1. M=4.7, SD=.8	1. M=4.4,SD=.9	[1]
2. Geneva		2.M=5.0,SD=NA.	2. M=4.7, SD=.8	2. M=4.5, SD=.9	
T-test		t(36)=-.4, *P*=.7. 95% CI: -1.819,1.224	t(94)=-.3, *P*=.8. 95% CI: -.431,.327	t(272)=-.6, *P*=.6. 95% CI: -.287,.155	
Qualitative ratings					
Physicians with comments					
1. Zurich	1. 18/225 (8.0)	**1. 37/406 (9.1)**	1. 74/356 (20.7)	**1.104/268 (38.8)**	[1]

2. Geneva	2. 13/185 (7.0)	**2. 1/437 (0.2)**	2. 22/331 (6.6)	**2. 67/280 (23.9)**	
Chi-squared-test	$\chi^2_{(1)}$=.1, *P*=.71	**$\chi^2_{(1)}$=38.2, *P*<.001**	$\chi^2_{(1)}$=1.5, *P*=.23	**$\chi^2_{(1)}$=14.8, *P*<.001**	
Average number of comments					
1. Zurich	1. M=1.1, SD=.3	1. M=2.4,SD=5.2	**1.M=2.8,SD=5.3**	**1.M=3.7,SD=5.5**	[1]
2. Geneva	2. M=1.4, SD=.7	2. M=1.0,SD=NA	**2.M=1.3, SD=.6**	**2.M=2.0,SD=2.1**	
T-test	t(16)=-1.4, *P*=.181. 95% CI: -.688,.141	t(36)=.3, *P*=.8. 95% CI: -9.327,12.084	**t(77)=2.4, *P*=.02. 95% CI: .270,2.752**	**t(142)=2.9, *P*=.005. 95% CI: .520,2.884**	

[1] Cell sizes too small to be analysed
