# Supplementary material for: Quantitative Ratings and Narrative Comments on Swiss Physician Rating Websites: Frequency Analysis
Source: J Med Internet Res. 2019 Jul 26;21(7):e13816. doi: 10.2196/13816 (PMC6688440; doi:10.2196/13816)
Supplement: Multimedia Appendix 2 [file jmir_v21i7e13816_app2.pdf]

Multimedia Appendix 2. Results of comparisons between specialities

Comparison	okdoc	docapp	medicosearch	google.ch	Overall
Quantitative ratings					
Identifiable physicians					
1. GPs<br>2.Specialists<br>Chi-squared-test / T-test	1. 230/526 (43.7)<br>2. 180/440 (40.9)<br>$\chi^2_{(1)}=.8$, *P*=.38	1. 464/526 (88.2)<br>2. 379/440 (86.1)<br>$\chi^2_{(1)}=.9$, *P*=.34	**1. 393/526 (74.7)**<br>**2. 294/440 (66.8)**<br>**$\chi^2_{(1)}=7.3$, *P*=.007**	1. 296/526 (56.3)<br>2. 252/440 (57.3)<br>$\chi^2_{(1)}=.1$, *P*=.755	1. M=2.6,SD=1.2<br>2. M=2.5,SD=1.3<br>t(900)=1.5, *P*=.14. 95% CI: -.038,.274
Rated physicians					
1. GPs<br>2. Specialists<br>Chi-squared-test / T-test	1. 38/230 (16.5)<br>2. 38/180 (21.2)<br>$\chi^2_{(1)}=1.4$, *P*=.24	1. 20/464 (4.3)<br>2. 18/379 (4.7)<br>$\chi^2_{(1)}=.1$, *P*=.76	1. 51/393 (13)<br>2. 45/294 (15.3)<br>$\chi^2_{(1)}=.8$, *P*=.38	**1. 122/296 (41.2)**<br>**2. 151/252 (59.9)**<br>**$\chi^2_{(1)}=19.1$, *P*<.001**	**1. M=.9,SD=.8**<br>**2. M=1.1, SD=.9**<br>**t(274)=-2.2, *P*=.03. 95% CI: -.413,-.020**
Average number of ratings					
1. GPs<br>2. Specialists<br>T-test	**1. M=1.7,SD=1.1**<br>**2. M=1.3, SD=.6**<br>**t(57)=2.1, *P*=.04. 95% CI: .016,.826**	1. M=3.0,SD=6.9<br>2. M=1.6,SD=1.5<br>t(36)=.9, *P*=.41. 95% CI: -2.014,4.792	1. M=2.1,SD=3.5<br>2. M=2.8,SD=5.7<br>t(94)=-.7, *P*=.47. 95% CI: -2.603,1.199	**1. M=2.8,SD=3.2**<br>**2. M=4.5,SD=5.5**<br>**t(249)=-3.2, *P*=.001. 95% CI: -2.776,-.675**	[1]
Average rating					
1. GPS<br>2. Specialists<br>T-test	NA	1. M=4.9, SD=.3<br>2. M=4.5,SD=1.0<br>t(19)=1.7, *P*=.11. 95% CI: -.105,.905	1. M=4.7, SD=.8<br>2. M=4.7, SD=.8<br>t(94)=-.02, *P*=.99. 95% CI: -.322,.317	1.M=4.4,SD=1.02<br>2. M=4.4, SD=.8<br>t(234)=-.5, *P*=.60. 95% CI: -.285,.166	[1]

Qualitative ratings					
Physicians with comments					
1. GPs	1. 17/230 (7.4)	1. 20/526 (3.8)	1. 51/51 (100)	**1. 66/293 (22.5)**	[1]
2. Specialists	2. 14/180 (7.8)	2. 18/440 (4.1)	2. 45/45 (100)	**2. 105/252 (41.7)**	
Chi-squared-test	$\chi^2_{(1)}$=.02,*P*=.88	$\chi^2_{(1)}$=.05, *P*=.82	NA	**$\chi^2_{(1)}$=23.1,*P*<.001**	
Average number of comments					
1. GPs	1. M=1.4, SD=.6	1. M=3.0,SD=7.0	1. M=2.1,SD=3.5	1. M=2.2, SD=2.5	[1]
2. Specialists	2. M=1.1, SD=.3	2. M=1.6,SD=1.5	2. M=2.8,SD=5.7	2. M=3.5, SD=5.4	
T-test	t(22)=1.7, *P*=.09. 95% CI: -.057,.620	t(36)=.8, *P*=.41. 95% CI: -2.014,4.792	t(94)=-.7, *P*=.48. 95% CI: -2.583,1.218	t(169)=-1.9, *P*=.062. 95% CI: -2.746,.065	

[1] Cell sizes too small to be analysed
